# European solidarity and “free movement of labour” during the pandemic: exposing the contradictions amid east–west migration

**DOI:** 10.1057/s41295-022-00287-4

**Published:** 2022-03-30

**Authors:** Dorota Szelewa, Michal Polakowski

**Affiliations:** 1 University College Dublin, Sheehy-Skeffington Building Belfield, Dublin 4, Ireland; 2 Poznan University of Economics and Business, Al. Niepodległości 10, 61-875 Poznan, Poland

**Keywords:** COVID-19, Labour mobility, Food industry, European Union, UK, Germany, Eastern Europe

## Abstract

COVID-19 regulations introduced in EU member states in 2020 meant serious restrictions for the free movement of persons, particularly workers. An ensuing gap in the supply of workers raised concerns of food shortages in the West. Governments in several EU member states enacted regulations to except the workers from restrictions facilitating their travel from Eastern Europe. In this study, we focus on EU-level responses to the COVID-19 crisis in relation to labour shortages in the food industry, and on the reactions in Germany and the UK. Firstly, referring to Schmidt (2020) and Wolff and Ladi (2020), we argue that the COVID-19 crisis placed the EU in a permanent emergency mode facilitating a quick response to enable labour mobility with less priority on the coordination of social rights. Secondly, the crisis exposed issues pertaining to working conditions, including housing and sanitation. Thirdly, differences between the reactions in Germany and the UK were consistent with the pre-existing trends in both countries. While a traditional emphasis on quality working conditions made it “appropriate” for the German government to initiate regulatory change, small-scale measures taken in the UK were directed towards maintaining an influx of migrant workers, rather than ensuring adequate working conditions.

## Introduction

COVID-19 regulations introduced in EU Member States meant serious restrictions for the free movement of persons, particularly workers, across the European Union (EU). Before countries such as Poland or Romania closed their borders to guard against entry of the virus, they allowed their citizens, often seasonal workers employed in Western European countries, to return to their home countries, organising extra flights from the most popular migrant destinations, such as London, Dublin, or Frankfurt. An ensuing gap in the supply of workers in the food sector almost immediately raised concerns of food shortages in the West. Governments in several EU member states enacted regulations to except these workers from travel restrictions and allow them to travel to their destinations, despite measures preventing the general EU population from travelling. Groups of Romanians and Bulgarians travelled (back) to Germany or the UK, possibly risking their own health.

Has the pandemic and ensuing labour shortages in Western European countries influenced or even transformed the regulation of working conditions, as well as public narratives about immigration? In this study, we focus on EU-level responses to the COVID-19 crisis in relation to labour shortages, as well as on the reactions in Germany and the UK. The UK was at the outset of leaving the EU, although it was still an EU member and hence subject to EU governance with respect to intra-EU labour mobility. While not focusing on the effect of Brexit per se, we examine developments in the UK in the context of the UK’s anticipated lack of EU membership. Although other sectors in Western Europe also suffered from an outflow of Eastern European labour, we focus on the food supply sector in this article^1^ because labour shortages in this particular sector constituted an emergency situation for both food producers and consumers. It became clear that food supply chains in several Western European countries would fall apart without Eastern European workers (Cosma et al. 2020).

This article examines how the COVID-19 pandemic represents “a revealing inflection point” (Rhodes 2021) that casts new light on both EU and Member States’ governance of mobility. Referring to Schmidt (2020) and Wolff and Ladi (2020), we present the reactions to COVID-19-related work shortages as embedded in the policies and discourses surrounding labour mobility, while also pointing to developments stemming from the EU’s “permanent emergency” mode. At the EU level, these developments were the consequence of an unusual mobilisation in a time of crisis. At the same time, this article highlights the EU’s limited interest in regulating this particular sphere, despite the high level of politicization and inclusion of COVID-19-related issues in EU governance (Schmidt 2020). Furthermore, while a traditional emphasis on quality working conditions made it “appropriate” for the German government to initiate regulatory change, only small-scale measures were taken in the UK, directed towards maintaining an influx of migrant workers, rather than ensuring adequate working conditions.

This article is structured as follows: First, we briefly discuss analytical approaches to East–West migration in the context of asymmetric EU integration and introduce a conceptual framework for analysing stability and change. This is followed by a brief background section. The next three sections analyse responses to the COVID-19 crisis in the context of labour shortages in the EU, Germany and the UK. The final part discusses the conclusions.

### Analysing the situation of mobile workers in the EU

Freedom of movement for workers coming from Eastern Europe was secured by EU Eastern Enlargement in 2004, whereupon Western European countries began to open their labour markets to Easterners, with the UK opening its labour market immediately and Germany completing this process in 2011. Millions of Eastern European workers migrated to the West seeking employment and better opportunities (Favell and Recchi 2009), leading to a “culture of migration” in countries such as Romania (Cosma et al. 2020). At the same time, many workers have been placed in a disadvantaged position, abused by employers, working under inadequate conditions, and often facing forced labour practices (Koroutchev 2020). These workers experience precarious conditions, have weaker bargaining power and often face hostility from the local populations for representing competition in terms of jobs, skills, and wages (Bogoeski 2020). Making an argument about the “moral economy of whiteness” in the UK, Garner (2012) referred to “Britishness” as frequently constructed at the local level against “the Others”, here, Eastern European migrants along with all other non-British people. This new “cultural” form of racism, according to Garner, goes beyond racial discrimination based on physical differences and extends to Eastern European migrant workers due not only to their “different” way of life and language issues, but also because of deep-seated resentment among local populations due to a perceived increase in competition in the labour market.

Studies also suggest that employers prefer mobile workers from Eastern Europe to domestic labour, treating ethnicity as a skill (Friberg and Midtbøen 2017). Romanian workers, who currently constitute the largest group of intra-EU migrants (Cosma et al. 2020), are willing to work for lower wages, have higher productivity, and, at the same time, are considered easy to replace by their fellow Romanians (García-Colón 2020). A the same time, the label “good workers” tends to be a trap because it generates expectations among employers that East Europeans work harder, including overtime, and are willing to engage in more difficult tasks (Baxter-Reid 2016).

Labour market segmentation, labour hierarchies, racialization and the labelling of Eastern European workers as “good workers” can be viewed in a larger context, wherein the emphasis in the EU integration process has been more on economic integration and less on the enhancement and/or reworking of the European social model or harmonization of social rights (Scharpf 2010). New post-economic crisis initiatives, including the Social Investment Package and the new directives within the European Pillar of Social Rights, increase the importance of EU socioeconomic integration (de la Porte and Heins 2016; de la Porte and Natali 2018; de la Porte et al. 2020). However, the current coronavirus situation brings to the forefront the discussion about how hierarchies in access to social rights are a consequence of inequalities in the labour market based on a worker’s country of origin (Bruzelius et al. 2017; Bruzelius 2018). In this context, Ferrera (2017) argued that a line of conflict existed between insiders and outsiders, referring to freedom of movement and its consequences. As the author argues, this tension has “a recognisable geographical dimension, running from East to West” (Ferrera 2017: 7), with the populations of the receiving countries showing hostility towards immigrants, who are “accused of ‘benefit tourism’ and held responsible for social dumping dynamics in terms of jobs and wages” (Ferrera 2017: 7). The geographical dimension of the conflict further calls for recognising a specific division of labour between the East and West of the EU, where the freedom of movement mostly refers to migrants from the East moving to work in Western labour markets.

As argued by Schmidt (2020), “EU governance in the COVID-19 crisis may very well result in paradigmatic change toward deeper European integration in some areas, incremental change in others, or even reversal toward disintegration in yet others” (Schmidt 2020: 1177). Building on this type of argumentation, this paper is interested in continuity and change, aiming to understand rather than explain the evolution of discourses and institutions. This paper is guided by a new institutionalist perspective, focusing on the most salient reactions to work shortages in Eastern Europe. The focus on change and continuity has often been at the centre of neoinstitutionalist theories. EU member states’ positions and strategies, as well as the evolution of EU institutions, were often perceived as path-dependent and set against a constellation of vested interests (Pierson 1996; Steinberg and Vermeiren 2016). Institutional complementarities support a given equilibrium until it is “punctuated” by an external shock that may trigger institutional change, or at least result in a potentially transformative shift in institutional dynamics (Campbell 1998). Among the three strands of institutionalism, historical institutionalism was often depicted as overly deterministic and unable to explain change. To escape this deadlock, scholars proposed either adherence to ideas as blueprints for institutional innovations and/or pointed to agency-based mechanisms, placing actors at the centre of the analysis (Mahoney and Thelen 2010). Other scholars stressed that policy-makers may mobilise certain discourses to justify policy change (Schmidt 2010, 2020), as discourse may enable or constrain different courses of action (Campbell 1998). Policy-makers may also be constrained by a certain constellation of vested interests or by existing ideas and discourses framing the interpretations of given phenomena (Schmidt 2010).

How can institutional evolution be detected, including the evolution or change in a paradigmatic discourse? On the one hand, we are interested in the question to what extent the COVID-19 crisis represents “a revealing inflection point–that casts light on their relative institutional strengths and weaknesses” (Rhodes 2021: 1537). On the other hand, because the analysis focuses on detecting possible institutional or policy shifts in response to the effects of the pandemic on Eastern European workers, we are interested in regulations (i.e., institutional change) and public perceptions/discourses. To assess institutional change, we examine regulations and policy responses at both the EU and Member State levels. In regard to public perceptions and discourses surrounding the role of Eastern European workers, we expect changes along the continuum from “paradigmatic change... i.e., a change in the dominant belief system” (Wolff and Ladi 2020: 1026) to smaller adjustments or partial shifts in perception. Instead of pointing to completely new developments only, we attempt to identify ongoing existing discourses and policies that constrain possible courses of action and that became salient and politicized due to the COVID-19 emergency (Schmidt 2019). We argue that while tensions around the free movement of labour that contribute to asymmetric integration continue to constrain the array of possible action, the salience of migrant workers’ plight increased during COVID-19, adding greater importance to already existing (or emerging) tools at the EU level, at agencies such as the European Labour Authority. In addition to an analysis of arguments regarding politicisation at the EU level, we turn to the Member State level to assess how EU membership (or a lack thereof) influences shifts in public discourse in EU Member States, bringing previously recognised, but thus far marginalised, issues to the surface.

To evaluate our hypothesis against developments at the EU level and in Germany and the UK, we conducted an empirical analysis focusing on the policies and practices that the EU and Member State governments introduced in response to labour shortages caused by COVID-19-related restrictions. In addition, we focus on the political salience of this topic as shown by public opinion surrounding Eastern European migrants during the pandemic. To trace legal and institutional/operational changes, we examined how various measures were introduced during different stages of pandemic management through document analysis, especially in regard to the EU. We used a structured and focused comparison, and employed qualitative analysis of the public debates in the UK and Germany. We reviewed the main internet outlets and newspapers in Germany and UK, searching for any print news, articles or television commentary that make reference to the food supply chain in the context of the pandemic and migrant workers. Additionally, we included secondary sources, such as commentaries and expert recommendations, including the first analyses of the situation of labour migration during the pandemic. We limited our timeframe for analysis to the year 2020, with some exceptions. We identified the most important arguments that were already present in public debates, relating to public perceptions of Eastern European migrants, pre-existing lines of conflict over intra-EU migration, health risks and working conditions, and tensions between EU citizenship and the sustainability of national production. The following section briefly outlines the context for the study.

### Background section

Following the EU Eastern Enlargement in 2004, Western European countries opened their labour markets to Eastern Europeans. The Posted Workers Directive further regulated employment conditions, allowing, among other things, an exception for posted workers in German meat factories to work under the same conditions as allowed in their countries of origin (Bogoeski 2019). The 2004 and 2007 enlargements were characterised by remarkable contradictions. On the one hand, only a handful of Member States (Ireland, the UK and Sweden) immediately opened their labour markets. On the other hand, both Ireland and the UK introduced policies that restricted access to social benefits (by establishing the Habitual Residence Condition and limiting access to social assistance, respectively).

Two selected cases–Germany and the UK–have a large share of immigrant labour; indeed, Germany and the UK had previously been used as cases for comparison (Bruzelius et al. 2017). In addition, these states rely heavily on intra-EU workers. According to the EU Commission’s most recent estimates, Germany and the UK hosted 25% of all EU-28 mobile workers in 2019. Romanians constituted the largest share of all economically active mobile EU citizens (23%), followed by Poles (16%) (European Commission 2020). In Germany, in August 2019, Polish and Romanian workers subject to social security contributions represented 19.7% and 17.6% of all EU mobile workers, respectively (followed by Italy, Croatia and Bulgaria) (Graf 2020). In the UK, Poles constituted the largest group of workers originating from Central and Eastern Europe.

In general, EU-28 mobile workers in agriculture (skilled occupations) displayed a significantly higher share of dependent employment (88%) than the native population (26%). Recent EU Commission estimates indicate that approximately 187,000 EU-mobile workers were employed seasonally in Germany in 2019, whereas only 6392 workers were posted for this purpose (European Commission 2022). In Germany, seasonal workers from Eastern Europe constituted approximately 67% of all seasonal workers in agriculture, forestry and fishing (Späth et al. 2018), while in the UK, this share increased to 98% (McGuiness and Garton Grimwood 2017).

Importantly, the enforcement of the working conditions in the UK has been seen as a multifaceted issue, as there are multiple agencies responsible for various aspects of employment, which presents coordination challenges (Tombs 2016). Furthermore, enforcement has been made more difficult due to the weakening power of trade unions, which used to be important partners in this task. In Germany, although multilevel enforcement of labour regulations exists, the governance of labour inspection is significantly more inclusive with respect to trade unions (Berlioz 2016). The International Labour Organisation’s statistics on labour inspectors show a decline of  − 2% in Germany between 2011 and 2018 and of  − 32% in the UK between 2011 and 2019. The dynamics of the decline in the number of inspections were similar in both countries during the respective periods ( − 25% in Germany,  − 27% in the UK). Yet, in Germany in 2018, 38 times more inspections (694,480) were conducted than in the UK (14,076). Finally, in Germany in 2018, there were 1.4 inspectors per 10 thousand workers, whereas this figure was only 0.4 in the UK. A report on the exploitation of labour in the UK argues that although migrants (including those from Eastern Europe) face a particularly high risk of exploitation, this is primarily due to the structural characteristics of the labour market, and not workers’ migration status per se (FLEX 2017).

In sum, migrant workers employed in sectors characterised by high seasonality face challenges arising from a lack of effective social security coordination, as well as from differences in labour protection and enforcement.

### The European Union

Reactions to the COVID-19 pandemic at the EU level followed in many respects the constitutional architecture of the Union and existing policy, although extraordinary measures emerged. The protection of freedom of movement was a central concern, and public health threats were not sufficient justifications for long-term border closures within the EU, as reflected in the Schengen Border Code. This may explain why border closures have been short-term (although recurring) (Thym and Bornemann 2020). With the emergence of the second wave of COVID-19 infections and various movement control modalities such as quarantine mandates, in October 2020, the Council developed a more consistent system of “traffic lights”.

While the Commission and Member States in general attached importance to reducing the mobility of individuals in Europe in the early phase of the pandemic, some categories of workers have been deemed “critical”, and their movement has been allowed nonetheless (OJEU 2020). In mid-March 2020, the Commission issued guidance in which it emphasised the importance of the mobility of “critical workers”, as well as the right of EU citizens to return to their home countries on a non-discriminatory basis. On 30 March 2020, in the Communication on Guidelines concerning workers’ exercise of free movement during the COVID-19 outbreak, the Commission included seasonal workers (referred to herein as workers or self-employed Member State citizens) as critical workers. In this way, seasonal workers joined a relatively narrowly defined category of EU citizens who are treated preferentially, “(i)n order to respond to labour shortages in these sectors [agriculture] as a result of the crisis” (p. 3).

As the public health crisis unfolded in Member States, more responses from EU institutions followed. The European Parliament resolution of 19 June 2020 on European protection of cross-border and seasonal workers in the context of the COVID-19 crisis called on the Commission and Member States to respect workers’ right to equal treatment, irrespective of national origin (European Parliament 2020). Furthermore, in its July 2020 Communication, the Commission called on Member States to ensure the execution of pre-existing health and safety guidance (European Commission 2020). Importantly, a notable gap emerged. While the document emphasises the importance of equal rights between natives and migrants and safeguards to protect seasonal and posted workers from non-EU countries, it also recognized that “[t]here is no Union act in place to guarantee accommodation conditions for other seasonal workers”, referring to intra-EU mobile workers. The Communication emphasised the need for more engagement by Member States but also acknowledged the role of the European Labour Authority,^2^ which gained heightened importance due to the pandemic.

The issue of intra-EU mobile workers certainly received more attention and salience. The activities of the Commission and the Parliament were met with the reaction of European trade unions, which expressed limited support for the EU’s initiatives and pointed out the need to develop binding standards on accommodation, transport, health and safety (ETUC 2020). Trade unions stressed that the “equal treatment approach” would be insufficient in sectors such as agriculture, where the either a lack of standards or their evasion is particularly pronounced (Martens 2020).

Later, the Council of the European Union Conclusions from October 2020 raised the issue of seasonal work, calling on Member States to fully apply existing regulations and to strengthen cooperation between the Commission and relevant stakeholders (Council of the European Union 2020). The conclusions reiterated that Member States remain responsible for health and safety and other working conditions.

The discussions on the employment conditions in the agricultural sector highlighted the structural characteristics of the sector. In negotiations concerning the new common agricultural policy, the Parliament in October 2020 proposed the introduction of the social conditionality clause (Foote 2021), which would make the payment of subsidies to agricultural operators conditional on the provision of adequate working conditions, as well as the satisfaction of other standards relating to, for example, public health or animal welfare. The social clause has been adopted and will become mandatory from 2025 (European Commission 2022).

Importantly, the prevailing theme of the communications from various EU bodies was that of a need to open internal borders so that seasonal workers could arrive in their destination countries. To a large extent, such an approach reflected the viewpoint of the Member States with labour shortages, which began to resonate at the EU level. A brief describing the preparation of MEPs (European Parliament 2021) illustrates this process. The brief provided an extensive overview of exploitative practices in agriculture while at the same time stating that.

“(t)he crisis has highlighted the critical role of migrant seasonal workers. As intra-EU borders were being closed, [these workers’] inability to reach host countries at the start of the harvest season in [the] EU for fruit and vegetables caused panic. Attempts to recruit workers locally to replace them often failed, as the work requires physical strength, endurance and speed that only experienced seasonal workers can provide; the long hours, low wages and hard-working conditions go some way to explain why a large part of EU agriculture relies on non-national labour” (ibid.: 10).

This passage emphasises four issues that emerged during the first year of the pandemic. First, the freedom of movement principle is of central importance and may be suspended only in a limited set of circumstances. Second, the inclusion of seasonal agricultural workers in the category of essential workers allowed them to circumvent the “hard” border closures among Member States. Third, the “soft” coordination of work and welfare issues was manifested again in the responses of the Commission and the Council. Fourth, there were positive developments regarding the European Labour Authority and Common Agricultural Policy. While possibly more effectively from the middle-term perspective, both the ELA and social conditionality in the CAP may have an impact on reducing the scale of substandard employment in the food supply sector. In sum, any expectations for a shift in the EU policy to provide more coordination in the regulation of working conditions were met to a limited extent. At the same time, the line of conflict surrounding the free movement of labour—which was less visible in the discussions within EU institutions, with the East–West dimension of the new initiatives almost entirely absent—was much more pronounced in the Member States, especially Germany and the UK.

### The German case

Long before the pandemic, the severity of migrants’ working conditions was well known to labour unions. Initiatives such as *Faire Mobilität* (Fair Mobility) aimed to enhance their working conditions and were partially state-funded, with cooperation and funding in part provided by the DGB—German Trade Union Confederation (Cosma et al. 2020). Similarly, gaps in enforcement of existing German labour laws and workplace standards, combined with the segmented character of the German labour market, were known to contribute to the situation of foreign workers (Baccaro and Benassi 2014; Durazzi et al. 2018) and led to the emergence of what Erol and Schulten called “a system of collective irresponsibility” (Erol and Schulten 2021: (2). Poor working conditions were linked to the employment status of most migrants in the meat-processing industry as agency, posted or subcontracted workers (Kuhlmann and Vogeler 2020). Moreover, in contrast to countries such as Denmark, France, and the Netherlands, minimum wages in German meat factories have been regulated only by company-level agreements; there is no industry-level agreement, and most establishments are not covered by collective agreements (Erol and Schulten 2021).

When the COVID-19 crisis arrived, Germany also introduced a series of mandatory measures to prevent the spread of disease, such as the closure of schools, nurseries, and universities, as well as shops, bars, and restaurants. On 22 March 2020, the government introduced an overall “contact ban” (Zajak et al. 2021). Germany introduced intra-European export embargoes on personal protection equipment along with France (Wieck et al. 2021). Almost immediately after travel restrictions were introduced, farmers declared labour shortages at the level of approximately 300,000 seasonal workers for the 2020 harvest (Deutsche Welle 2020c). An online platform was created to advertise jobs quickly for local employees, but these only attracted 16,000 applicants, who usually had no experience harvesting asparagus. Other actions aimed to mobilise the domestic labour force, including initiatives such as “Das Land Hilft” (daslandhilft.de, i.e., “The Country Helps’) and “Fridays for Future” in Hessen and Bavaria.

However, it was very difficult to recruit the domestic labour force, which was attributed to an unwillingness among domestic labour, and workers from Eastern Europe were found to be more physically fit than German workers. According to farmer Ernst-August Winkelmann: "(l)ong-term unemployed Germans are no longer used to working for their money," (…) "Six weeks of hard physical labour appears to be so unattractive to Germans that none of them lasts the course. Also, the prospect of facing unemployment again after the harvest discourages some of them." (Deutsche Welle 2020b). Other farmers expressed a preference for Eastern European workers, referring to them as “reliable, punctual and do good work," while "most of (the Germans) do not even show up" (Deutsche Welle 2020c). Workers from Eastern Europe were also perceived as “strong, frugal and working hard (…). They provide a humiliating example for our own spoiled workers, who demand much, but deliver little." (ibid.). Furthermore, the Federal Labour Office spokesperson perceived the hiring of domestic workers as economically disadvantageous, as "there could be economic disadvantages for the employer if we send over unmotivated workers, and we do not want to do that” (ibid.).

On April 2, 2020, the German Ministry for Food and Agriculture (BMEL) announced a travel strategy to overcome the labour shortage (Lakner 2020). In response, the Romanian Ministry of Interior announced on 4 April 2020 that seasonal harvest workers would be given permission to leave the country to work abroad. More than 120,000 workers were transported from Romania to Germany during April and May 2020 by Eurowings, and were welcomed at the Frankfurt-Hahn airport by the German Minister of Agriculture and with chocolate Easter eggs (Miesenberger 2020). The workers had been allowed to take a direct flight from Romania, even though it was clear that maintaining physical distancing was impossible (Deutsche Welle 2020c). Photos from Cluj-Napoca airport show distressed Romanians queuing to enter the airport, crowded, and clearly not maintaining social distancing (EUobserver 2020). Migrants reported that the main reason they had agreed to take this position was economic necessity, including the loss of their own source of income in Romania due to lockdown measures and other types of pandemic-related restrictions (Deutsche Welle 2020a). Romanian migrants employed to harvest asparagus in Germany report extremely difficult working conditions, including 10-h shifts seven days a week, with low wages, often allowing for a savings of only €1800 after three months (Deutsche Welle 2020a).

After arriving in Germany, Romanian workers complained about poor working conditions and hygiene and about social distancing rules not being respected, they were forced to work 14–16 h per day. The German-Romanian Association for Integration and Migration SGRIM sent a letter to the Romanian government mentioning these concerns, as well as cases in which workers were not afforded the protection of an employment contract after their arrival in Germany: “their [workers’] rights are not respected and (…) they are treated like slaves” (Neagu 2020). In addition to the continued exploitation of these workers, the employers replaced (promised) COVID-19 tests with temperature checks (Paul 2020). Perhaps the most salient case was a 57-year-old Romanian worker who died and was diagnosed with COVID-19 post-mortem after he reported feeling unwell; but his employer had refused to take him to the hospital (Andriescu 2020). At that point, some German politicians started to openly criticise the companies employing Eastern European workers, calling them “scandalous and irresponsible in every respect” (Eddy 2020 as quoted by Paul 2020: 255). They were joined in their criticism by local civil society organisations, churches and trade unions, which provided support to migrants (Deutsche Welle 2020a).

Health risks were even greater in slaughterhouses, which experienced several outbreaks of the COVID-19 virus that infected thousands of workers. As reported by trade unions, 80% of all workers employed in the meat industry (almost 100,000) were from Eastern Europe (Witting 2020); almost all infected workers were from Poland, Bulgaria, Romania and other countries of the region. Poor living and working conditions were to blame, as well as “working hour violations, inadequate health and safety at work and disgraceful living accommodations" (Witting 2020 quoting Anja Piel, a board member with the German Trade Union Confederation). Even though employees were risking their own health and becoming infected, they were blamed for spreading the virus (Bogoeski 2020).

Almost immediately after additional resources were used to attract more local workers to harvest asparagus, it became clear that the asparagus farmers needed employees with certain skill sets and experience in harvesting. As argued by Joachim Rukwied, president of the German Farmers’ Association (DBV), “refugees and unemployed workers were an imperfect substitute as they often lacked the relevant expertise” (Buck et al. 2020). Interestingly, German employers also feared competition from other Western European countries in attracting Eastern European workers, urging the German government to act quickly.

Among other arguments, European solidarity was raised among the migrants themselves—one of the Bulgarian workers interviewed on site of the Tönnies meat processing factory complained that “[w]e're European as well. We have rights. You cannot put us behind a fence” (Lee 2020). This is consistent with Paul’s (2020) argument that these workers had an active role in loosening the restrictions so that they could be employed. But apart from lobbying to reopen the borders and for recognition as essential workers, the mobilisation of Eastern European workers was to address issues of health and safety, as well as working conditions.

The government reactions were initially aimed at facilitating the mobility. First, the regulations were aimed at enabling Romanian and Bulgarian workers to be transported to Germany despite closed borders. Other immediate measures included extending the period of exemption from social security contributions for employers from 70 to 115 days, which contributed to the precarious situation of Eastern European workers. Although not a regulation, the *Faire Mobilität* also established the COVID-19 info-hotline for Eastern European workers (*Corona-Hotline für Osteuropäische Beschäftigte*), through which social and labour rights experts provided comprehensive information and direct support. Most often, the reasons for contacting the hotline involved wage issues or wage theft, lack of information, health and safety concerns or violations of social rights (Eurofound 2020).

However, the most crucial change at the regulatory level was the introduction of a ban on outsourcing labour in the fresh vegetable and meat sectors. The Occupational Safety and Health Inspection Act, 2020 (Arbeitsschutzkontrollgesetz) provides a wide-ranging body of statutory regulation, including a ban on contract work in the industry and restrictions on the use of temporary-agency employment. The measure was adopted almost as a direct response to the outbreak of COVID-19 in meat factories, which exposed the exploitative working conditions that had been a common feature of the meat industry’s operation for years. In sum, even though the salience of this issue had been triggered to a large extent by the epidemiological threat that overcrowded workplaces posed to German society, it may eventually contribute to policy developments and operational changes that improve migrants’ working conditions.

### The UK case

For a long time, labour shortages in the UK had been met by the migration of workers from Eastern European countries and non-EU third countries. The visa system of the UK had been skewed in favour of highly skilled non-European immigrants, with the Seasonal Agricultural Workers Scheme (SAWS) phased out. The declining importance of the SAWS could be attributed to the workers inflows from Eastern European countries that joined the EU (especially Romania and Bulgaria). In 2017, 80,000 workers were employed in fruit picking and vegetable harvesting, while 13,000 were required at the pre-Christmas peak in the poultry industry (National Farmers Union 2020). In this sense, one can talk about a growing substitution of non-European workers, first by EU8, and then by EU2 (Bulgaria and Romania). On some farms, workers originating from EU2 countries constituted 95% of workers. Yet, one of the most dominant public narratives about migrants in Britain has pictured Eastern European migrants as “unwanted, low-skilled individuals who sponge off of the UK benefits system” (Andriescu 2020).

Discussions regarding labour shortages in the agricultural sector in the UK precede the COVID-19 crisis. In 2016 and 2017 organisations representing the sector warned that inaction would lead to “food rotting in the fields”. The report published by the House of Commons in 2017 summarises this discussion and delivers some interesting insights into the perception of labour shortages by various stakeholders from the sector (House of Commons 2017).

First, agricultural organisations noted significant problems in recruiting the domestic workforce, which had to do with the arduous and poorly paid nature of agricultural work. However, arguments about British workers were raised by one of the experts: “It was just that people do not want to do the work I mentioned before [because it comes with] rural locations and unsociable hours. Quite frankly, they could find jobs that they wanted to do that would be in easier and more agreeable conditions for the same amount of money” (House of Commons 2017).

Second, foreign workers proved more difficult to recruit, which had to do with their preference for work in other sectors, less favourable exchange rate or “increased living standards in Eastern Europe” (ibid.). Furthermore, individuals from Eastern Europe did not feel welcome in the UK. In the words of representative of British agriculture:

traditionally, UK has been sourcing workers from the region [Eastern Europe] (…) to a degree we are an international marketplace for the kind of seasonal labour we are talking about. If you look across Europe, southern Spain’s primarily manual labour comes from Morocco. For the low countries it very often comes from Turkey. These other countries have their established sources, if you like. We traditionally had established sources in central and eastern Europe. (ibid.).

In March 2020, agriculture operators expressed their concerns, stating that the sector needed 90,000 workers in a matter of weeks. While some farms had already started using charter flights in March/early April, large organizations, such as recruitment agencies, called for government guarantees that migrant workers would be admitted. Eventually, this option ceased to be viable, as several countries of the CEE closed their borders and suspended flights.

Similar to Germany, but perhaps with a stronger emphasis, the first reaction of British employers was to attract domestic workers, often citing national values and national sustainability of production as the reason. Temporary work agencies launched domestic campaigns to attract British workers, appealing to their sense of national belonging, as in the *Feed the Nation* action (Telegraph 2020). The Telegraph article depicted the work in the following terms: “Think of it as your daily dose of exercise, extended over some hours (and you’re helping to prop up the country’s food supply, while you’re at it)” (ibid). A representative of the National Farmers Union (NFU) stated, “Government must ensure that British poultry meat, and the quality it represents, stays affordable and available for all. Losing control of ourselves as a nation would penalise British food producers at a time when we should be taking matters of food security into our own hands” (British Poultry Council 2020).

A month later, a flagship initiative, backed by the government and called “Pick for Britain”, was announced. The representative of the NFU commented on the campaign:

The Pick for Britain campaign was a great initiative, and many have suggested we could continue to recruit a domestic workforce for the coming 2021 season and beyond. While there was a fantastic response from Brits to the call for domestic workers this year in extraordinary circumstances, we see from the survey results that they only made up 11% of the workforce. Seasonal work on farms simply isn’t a viable solution for many (National Farmers Union 2020).

The representative pointed out that recruitment of non-EU workers is crucial, saying that “the frustrating thing is this situation is easily solved with the implementation of a seasonal worker scheme, building upon the pilot scheme that has already operated successfully for the past two seasons” (ibid.). The demand for foreign workers was contrasted with the sacrifices the British would have to make: “what we’re asking of [UK workers] is huge. In reality, it means people needing to work in very rural areas, away from their homes and families, where they will only have guaranteed work for between three and six months” (Buck et al. 2020).

Attempts to replace migrant workers with domestic labour had been very difficult from the start. The COVID-19 pandemic revealed to the general public the dependence of British agriculture on foreign workers, especially those from Romania and Bulgaria. The debate pointed out that these workers have been an essential component of the sector. It also became apparent that domestic workers were not willing to accept this is type of work due to its characteristics (long hours, low pay, intensity) and context (rural areas, living in employer-provided dorms). Another important point was raised: seasonal work in agriculture requires a certain set of skills. British employers insisted that they needed migrant workers to train British workers.

While approximately 36,000–50,000 Brits registered to participate in the action “Feed the Nation”, only 6000 participated in interviews, approximately 1000 were offered a job, and as few as 112 individuals accepted contracts. Similarly, while anecdotal evidence points to limited success, no official data have been published, but it was agreed that the *Pick for Britain* campaign appeared to be a failure. One of the agricultural managers commented on British workers: “people discovered how tough the job was, with 5 am starts, working in all weathers and a rate of picking that needed to be cost effective. We need people who are productive” (Belger 2020). Among the reasons provided were that Brits “cannot commit to 40 h a week, some can only commit for a few weeks whereas some roles can be full time for eight weeks and some can be up to six months’” (The Guardian 2020). While working conditions and health risk almost immediately became the most salient issues in Germany in the public debate, these issues had not been as important in the public debate in the UK, where the focus of the public debate was more on sustaining food production.

In June 2020, the government introduced a 14-day self-quarantine requirement for all individuals arriving in the UK. However, some workers in the food industry were exempt from this requirement. In practical terms, this meant that workers could start work immediately while following the social distancing rules. The self-quarantine period was subsequently reduced to ten days. In November 2020, the National Farmers Union announced that it had successfully lobbied the government to ease quarantine requirements for migrants arriving to work in the poultry industry. This regulation allowed migrant workers to work immediately upon arrival in England during the 14-day quarantine period. But apart from these ad hoc measures, there were hardly any laws at the national level aimed at responding to the situation on a systemic level, other than calls to facilitate the arrival of seasonal workers from Eastern Europe and to extend the Seasonal Workers Scheme for non-EEA citizens.

In sum, the dependence of the UK’s farming sector on foreign seasonal labour became particularly apparent when the external borders of both the EU and the Member States were closed in response to the COVID-19 pandemic, significantly limiting labour mobility. Additionally, the COVID-19 pandemic also showed another iteration of the Brexit paradox involving the use of anti-migration rhetoric while at the same time relying on foreign workers to produce “cheap British food”. And, as demonstrated below, even in the face of “harvest rotting in the fields”, there was a complete failure to mobilise the domestic workforce. Finally, the COVID-19 pandemic served as a catalyst for voicing concerns about a post-Brexit migration policy.

## Conclusions

The goal of this article was to analyse the impact of labour shortages and threat of food crises caused by the pandemic on the EU’s role in coordinating the working conditions of mobile workers with Member States and on public policy and debate surrounding immigration in Germany and the UK. We relied on Schmidt’s (2019, 2020) and Wolff and Ladi (2020) arguments explaining how the COVID-19 crisis placed the EU in a permanent emergency mode facilitating a quick response. The EU was mostly motivated by the emergency in the food sector to facilitate labour mobility and placed less priority on the coordination of social rights. Although the COVID-19 emergency shed new light on the salience of agencies like the European Labour Agency, this agency could have been utilised for more concrete and immediate actions, such as pressuring national labour inspectorates to issue “European certificates” in health and safety (Petmesidou and Guillén 2020). Both the UK and Germany experienced similar obstacles in replacing Eastern Europeans with domestic workers. The situation has brought to light preexisting and partly known issues pertaining to working conditions, including housing and sanitation, which have suddenly become more salient due to the health risks to employees and the public. In both countries, an emphasis was placed on the specific suitability of Eastern European workers for jobs in the food industry.

Apart from characterising the reaction to COVID-19-driven public narratives and policies, we point to differences between Germany and the UK. While the discourse in Germany was much more focused on the issues of working conditions and health risks, it also generally referred to the larger context of the EU. At the same time, the dominant narrative in the UK was about the sustainability of production and perseverance of the “British” food industry. At the discourse level, there was a smaller emphasis on working conditions and health risks in the UK than in Germany, where the outbreak of COVID-19, especially in meat processing factories, became the most important issue discussed in the debates surrounding the reform to ban the outsourcing of labour in the industry. Another difference was a much stronger emphasis in the UK on the national sustainability of food production and the absence of reference to the European context, most likely due to Brexit. Although references to EU citizenship were also not very common in the German debate, such concerns were raised, among others, by the workers themselves. Finally, while the measures undertaken in the UK mostly sought to facilitate the movement of Eastern European workers to Britain, in Germany, the discussion led to the introduction of reforms at the federal level. We further argue that these differences are consistent with the pre-existing trends in both countries. First, there was a lower commitment to securing quality working conditions in the UK than in Germany, where the issue of the inferior working conditions of Eastern European migrants was already recognised, although it was not (yet) at the centre of public debate. Second, the traditional emphasis on quality working conditions in the (continuing) context of the EU made it “appropriate” for the German government to initiate regulatory change. Measures taken in the UK were small scale and directed towards maintaining an influx of migrant workers rather than improving working conditions. Although this requires further research, the division of labour between Eastern and Western Europe, with the specific role of Eastern European migrants as essential and non-replaceable workers, is another iteration of the dominance of economic interests over “Social Europe”.

## Footnotes

1 The food supply sector consists of food production, food manufacturing (70% of all employment), food retail and wholesale, and smaller production/trade activities.

2 The European Labour Authority is an EU agency established in 2019 and will become fully operational in 2024.
